# Supplementary material for: Bioinformatics Analysis of the Molecular Networks Associated with the Amelioration of Aberrant Gene Expression by a Tyr–Trp Dipeptide in Brains Treated with the Amyloid-β Peptide
Source: Nutrients. 2023 Jun 13;15(12):2731. doi: 10.3390/nu15122731 (PMC10305223; doi:10.3390/nu15122731)
Supplement: Supplementary file 1 [file nutrients-15-02731-s001.zip › nutrients-2381356-supplementary.pptx]

## Slide 1
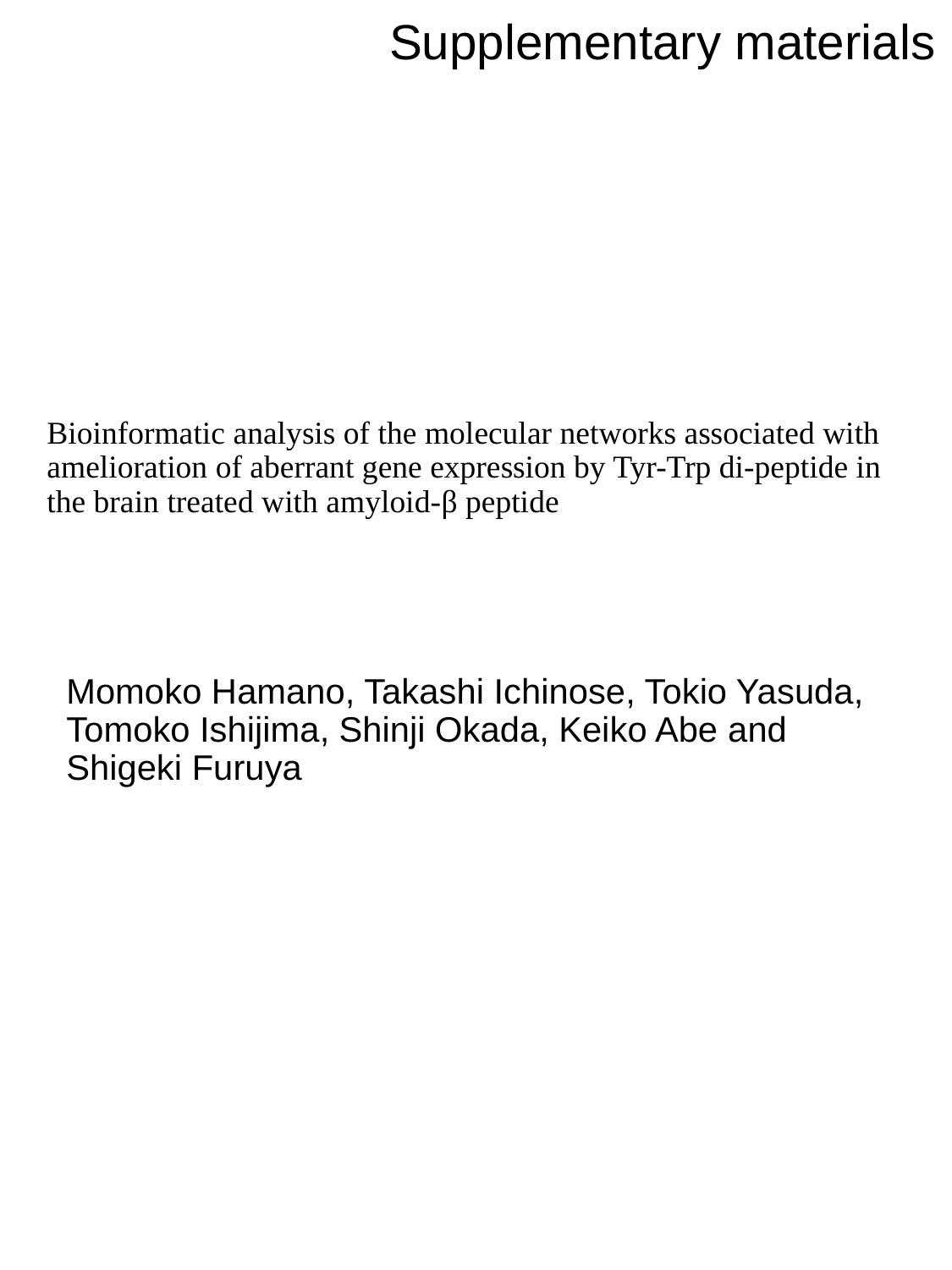

Supplementary materials
Bioinformatic analysis of the molecular networks associated with amelioration of aberrant gene expression by Tyr-Trp di-peptide in the brain treated with amyloid-β peptide
Momoko Hamano, Takashi Ichinose, Tokio Yasuda, Tomoko Ishijima, Shinji Okada, Keiko Abe and Shigeki Furuya

## Slide 2
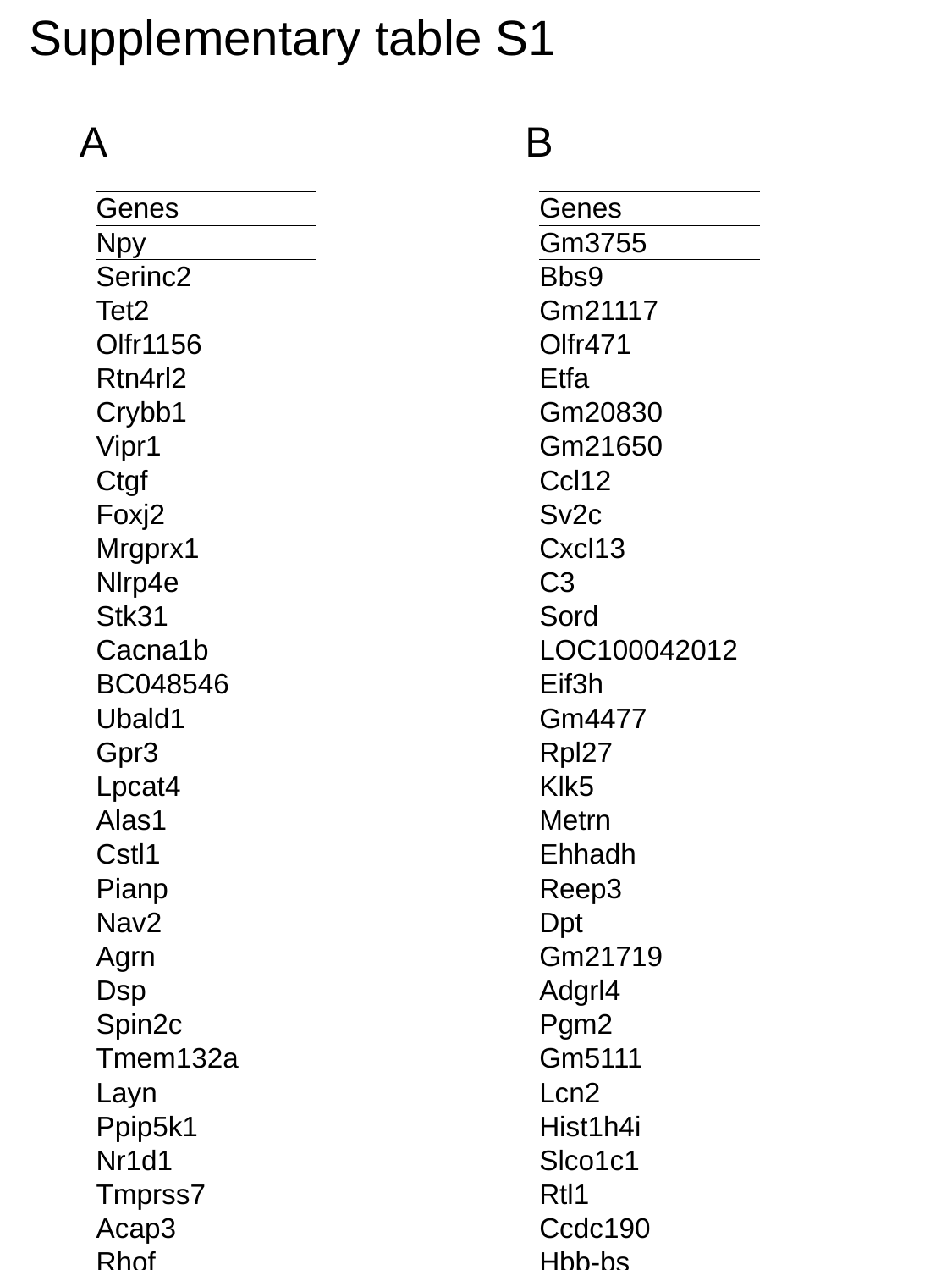

Supplementary table S1
A
B
| Genes |
| --- |
| Npy |
| Serinc2 |
| Tet2 |
| Olfr1156 |
| Rtn4rl2 |
| Crybb1 |
| Vipr1 |
| Ctgf |
| Foxj2 |
| Mrgprx1 |
| Nlrp4e |
| Stk31 |
| Cacna1b |
| BC048546 |
| Ubald1 |
| Gpr3 |
| Lpcat4 |
| Alas1 |
| Cstl1 |
| Pianp |
| Nav2 |
| Agrn |
| Dsp |
| Spin2c |
| Tmem132a |
| Layn |
| Ppip5k1 |
| Nr1d1 |
| Tmprss7 |
| Acap3 |
| Rhof |
| Plk3 |
| Etv5 |
| Nr4a2 |
| Txndc8 |
| Man1c1 |
| Prrt2 |
| Bhlhe40 |
| Fbrs |
| Tmem39b |
| Olfr1039 |
| Zfp691 |
| Olfr594 |
| Rab3b |
| Egr3 |
| Cited4 |
| Krtap9-3 |
| Rcor3 |
| Adgrb1 |
| Nop2 |
| Ifna11 |
| Inha |
| Cldn34b3 |
| Gm10912 |
| Ncoa3 |
| Fbxo17 |
| Slc19a2 |
| Rimbp2 |
| Vmn2r85 |
| Tcstv1 |
| 1700007K09Rik |
| Vmn1r57 |
| Nagpa |
| Foxo6 |
| Ccdc129 |
| Stambpl1 |
| Desi2 |
| Proser1 |
| Osbpl3 |
| Atxn2 |
| Wdr88 |
| Tgfb1i1 |
| Pdgfb |
| Efna3 |
| Dnase1l2 |
| Car10 |
| Slc7a6 |
| Exo1 |
| Spns3 |
| Plk2 |
| Tdg |
| Cacna1g |
| Kdm8 |
| Prdm8 |
| Olfr1223 |
| Tbr1 |
| Akap4 |
| Cacna1i |
| Olfr1496 |
| Cdkn1a |
| Neurod2 |
| Galnt9 |
| Coro1a |
| Grm2 |
| Tcerg1 |
| Nr4a3 |
| Ptger1 |
| Tnrc6c |
| Vgf |
| Fam13c |
| Plxdc1 |
| Rhot1 |
| Otud3 |
| Helq |
| Arhgef19 |
| Prrt1 |
| Rpp25 |
| Fam174b |
| Myadm |
| Eml5 |
| Phlda1 |
| Slc35c1 |
| Sh3bgrl3 |
| BC049702 |
| Lpar5 |
| 4833420G17Rik |
| Mef2d |
| Rgsl1 |
| Sprn |
| Osbp2 |
| Cdca4 |
| Vps9d1 |
| Csn1s2a |
| Rab35 |
| Ccl25 |
| Ackr1 |
| Spice1 |
| Prpf18 |
| Dnm1 |
| Vps37b |
| Pcdhb12 |
| Tagln3 |
| Prr12 |
| Nvl |
| Slc22a12 |
| Nfib |
| Rspo2 |
| Uck2 |
| Coro6 |
| Trio |
| Olfr1008 |
| Tmem201 |
| Fam65a |
| Otub2 |
| Gm10845 |
| BC030499 |
| Prl7a1 |
| Nabp1 |
| Hdx |
| Baiap2l1 |
| Prr36 |
| Smarcd2 |
| Armc6 |
| Gm16833 |
| Cidec |
| Olfr473 |
| Wnt10a |
| Pias1 |
| Mamld1 |
| Siah2 |
| 3110035E14Rik |
| Cck |
| Sphk2 |
| Lmtk3 |
| BC048502 |
| Genes |
| --- |
| Gm3755 |
| Bbs9 |
| Gm21117 |
| Olfr471 |
| Etfa |
| Gm20830 |
| Gm21650 |
| Ccl12 |
| Sv2c |
| Cxcl13 |
| C3 |
| Sord |
| LOC100042012 |
| Eif3h |
| Gm4477 |
| Rpl27 |
| Klk5 |
| Metrn |
| Ehhadh |
| Reep3 |
| Dpt |
| Gm21719 |
| Adgrl4 |
| Pgm2 |
| Gm5111 |
| Lcn2 |
| Hist1h4i |
| Slco1c1 |
| Rtl1 |
| Ccdc190 |
| Hbb-bs |
| LOC100042443 |
| Dhx37 |
| Pfdn4 |
| Gm21921 |
| Uts2r |
| Col5a3 |
| LOC100039905 |
| Gm14632 |
| Gm3043 |
| LOC100041897 |
| Rbm4b |
| Vat1 |
| S100a16 |
| Rps27a |
| LOC100862113 |
| Gm10319 |
| 1810037I17Rik |
| Hsdl2 |
| Micu2 |
| Rbm4 |
| Mcm2 |
| Plekhb1 |
| Gm20871 |
| Rpn2 |
| Nme2 |
| Khnyn |
| Gm21800 |
| Pcdh17 |
| Trpm3 |
| Stac |
| Nhlrc2 |
| Zeb1 |
| C2 |
| Rbp1 |
| Saa2 |
| Gm20826 |
| Wdr38 |
| Mesdc2 |
| Gm10323 |
| Ddit3 |
| Hbb-bt |
| Svopl |
| Npm2 |
| AI607873 |
| Tkt |
| 4933407L21Rik |
| Cml3 |
| 1700067P10Rik |
| Gabrg1 |
| Pycard |
| S100a4 |
| Osbpl9 |
| Cd59a |
| Rdh5 |
| Nlrp4a |
| Angptl4 |
| Cbx7 |
| Olfr1002 |
| Gm21518 |
| Gm9602 |
| Gm20736 |
| Dnali1 |
| Sgms2 |
| Gm8841 |
| Rasl2-9 |
| Rhox2e |
| Oaz1 |
| Prorsd1 |
| Gm20806 |
| LOC100042279 |
| LOC100862223 |
| Trdn |
| Zfp640 |
| Fam114a1 |
| Vmn1r202 |
| Gm21943 |
| Slc15a3 |
| Cyyr1 |
| Gm21732 |
| Atp6v0e |
| Gm20888 |
| Gm20917 |
| Gm10408 |
| Stk11 |
| Dbi |
| BC048679 |
| Nme3 |
| Mospd3 |
| Cyp2j11 |
| Skint8 |
| Tm2d1 |
| Hyal2 |
| Upk1a |
| Acadl |
| Ctnnbip1 |
| Olfr1155 |
| Stpg1 |
| Ptrf |
| Hebp1 |
| Acaa2 |
| Rpl38 |
| Clec7a |
| Oat |
| Sly |
| Shroom3 |
| Zak |
| A730020M07Rik |
| Klhl4 |
| Phgdh |
| Cpb1 |
| Arl3 |
| Zkscan4 |
| H2afj |
| Rassf4 |
| Brms1 |
| LOC105242925 |
| Heph |
| Igfbp2 |
| Ptgfr |
| Endov |
| P2rx5 |
| Grhl3 |
| P3h1 |
| Fuom |
| Rmrp |

## Slide 3
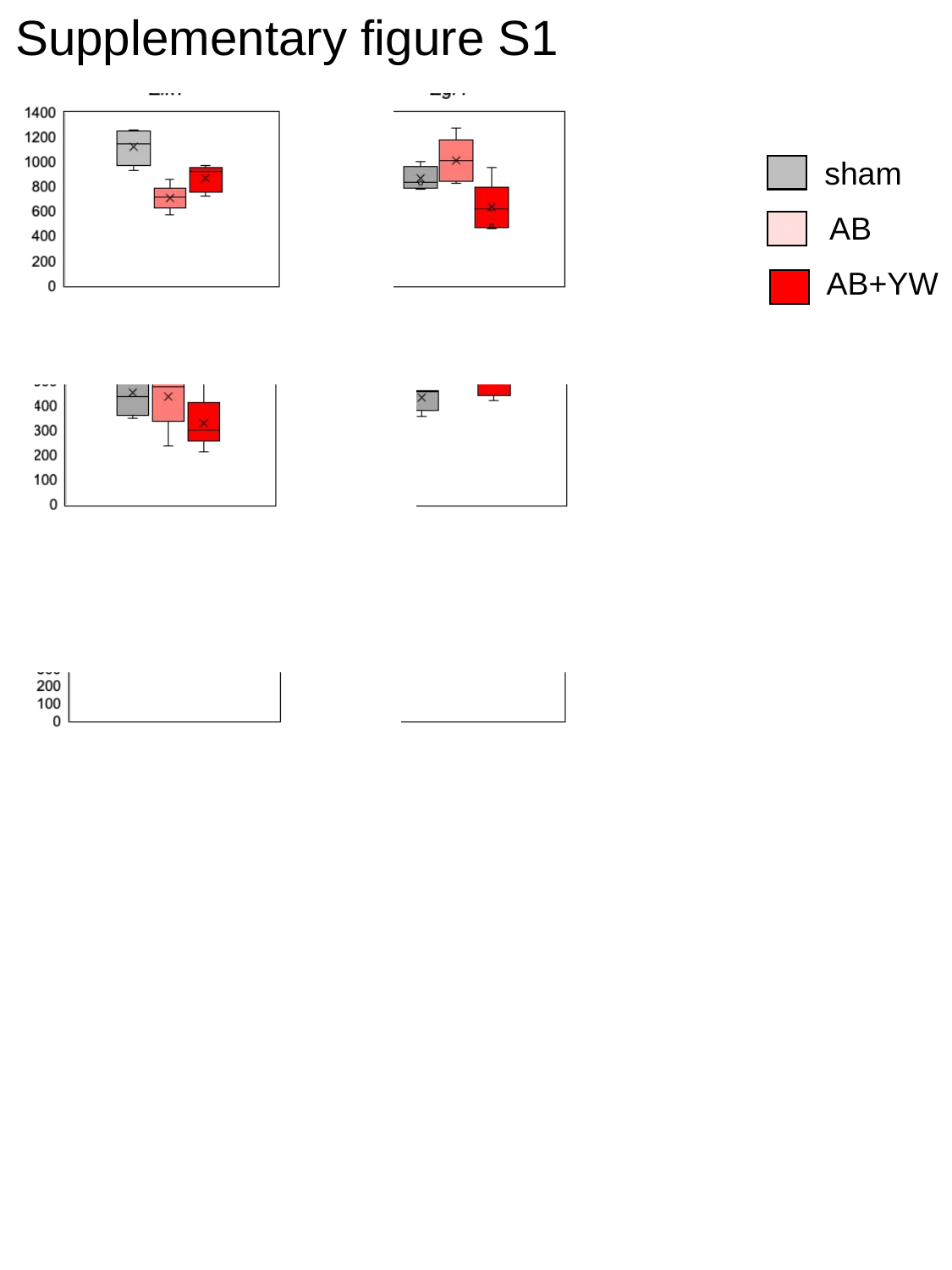

Supplementary figure S1
sham
AB
AB+YW

## Slide 4
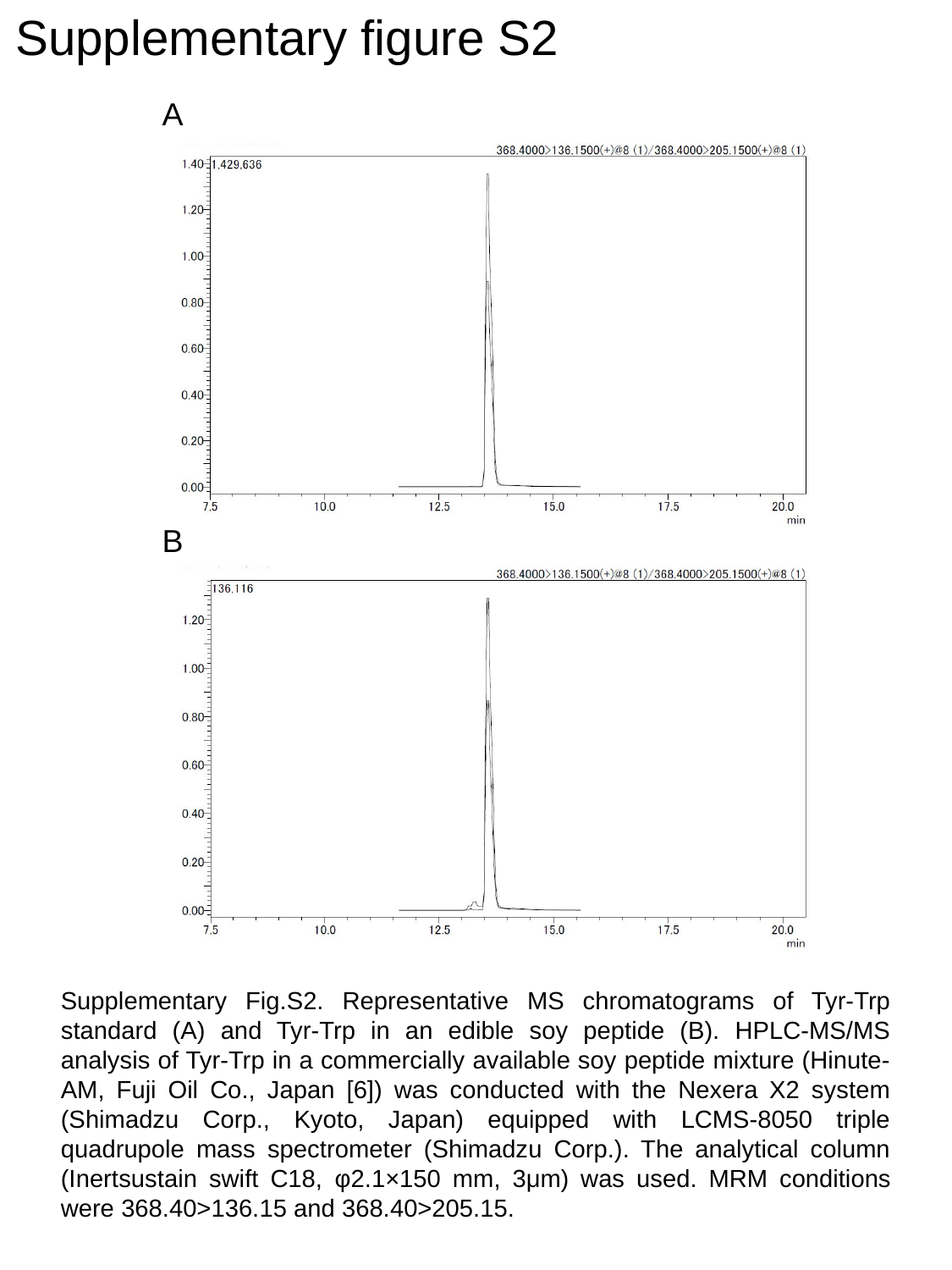

Supplementary figure S2
A
B
Supplementary Fig.S2. Representative MS chromatograms of Tyr-Trp standard (A) and Tyr-Trp in an edible soy peptide (B). HPLC-MS/MS analysis of Tyr-Trp in a commercially available soy peptide mixture (Hinute-AM, Fuji Oil Co., Japan [6]) was conducted with the Nexera X2 system (Shimadzu Corp., Kyoto, Japan) equipped with LCMS-8050 triple quadrupole mass spectrometer (Shimadzu Corp.). The analytical column (Inertsustain swift C18, φ2.1×150 mm, 3μm) was used. MRM conditions were 368.40>136.15 and 368.40>205.15.
